# Genetic and Clinical Factors Influencing Congenital Anomalies of the Kidney and Urinary Tract in Children: Insights from Prenatal and Postnatal Assessments

**DOI:** 10.3390/biomedicines12081798

**Published:** 2024-08-08

**Authors:** Hülya Gözde Önal, Hülya Nalçacıoğlu, Demet Tekcan Karalı, Mesut Önal, Beytullah Yağız, Meltem Necibe Ceyhan Bilgici

**Affiliations:** 1 Department of Pediatric Nephrology, Ondokuz Mayıs University Faculty of Medicine, 55270 Samsun, Turkey; 2 Department of Gynecology and Obstetrics, Ondokuz Mayıs University Faculty of Medicine, 55270 Samsun, Turkey; 3 Department of Pediatric Surgery, Ondokuz Mayıs University Faculty of Medicine, 55270 Samsun, Turkey; 4 Department of Radiology, Ondokuz Mayıs University Faculty of Medicine, 55270 Samsun, Turkey

**Keywords:** CAKUT, kidney, malformations, nephrogenesis, risk factors, outcomes

## Abstract

Congenital anomalies of the kidney and urinary tract (CAKUT) significantly contribute to pediatric morbidity, often necessitating ureterorenal surgery. This study explored the relationship between genetic mutations, renal surgery requirements, and prenatal, postnatal, and parental risk factors in children with CAKUT. A retrospective analysis of 651 children diagnosed with CAKUT included patient demographics, parental risk factors, ultrasound findings, genetic mutations, and surgical incidence. Antenatal ultrasounds showed normal findings in 32.1%, hydronephrosis in 46.9%, and other abnormalities in 21% of cases. Surgical intervention correlated with higher hydronephrosis reduction than non-intervention. Genetic mutations were identified in 5.4% of cases, with 24.6% requiring surgery. Low neonatal birth weight (odds ratio [OR] = 0.98, *p* < 0.001), advanced maternal age (OR = 1.06, *p* < 0.001), and postnatal abnormal ultrasound findings (OR = 2.62, *p* < 0.001) were associated with increased genetic mutation risks. Antenatal hydronephrosis (OR = 3.85, *p* < 0.001) and postnatal urinary tract infections (OR = 4.85, *p* < 0.001) increased the likelihood of surgical intervention. Neonatal birth weight, maternal age, and postnatal ultrasound findings were identified as independent risk factors for genetic mutations, while no significant link was found between these genetic factors and the need for surgery. Surgical needs were associated with urinary tract infections and antenatal hydronephrosis, indicating that timely surgical intervention may benefit these patients.

## 1. Introduction

Congenital anomalies of the kidney and urinary tract (CAKUT) include a broad spectrum of structural abnormalities that occur during fetal development [1]. These anomalies are among the most common congenital malformations, affecting 3–7 out of 1000 live births, and a leading cause of chronic kidney disease and renal failure in children [2]. The causes of CAKUT are complex and multifaceted, involving genetic mutations, maternal factors, fetal environmental exposures, and obstructive factors that disrupt normal kidney and urinary tract development [3].

Advances in genetic research have identified several genes associated with CAKUT, providing insights into its hereditary patterns and molecular mechanisms. Key genes implicated include transcription factors (e.g., *PAX2* and *HNF1B*), signaling molecules (e.g., ROBO2), and components of the extracellular matrix (e.g., *FRAS1* and *ITGA8*) [4,5,6]. Chr.17q12 deletion (renal cyst and diabetes syndrome), Chr.22q11.2 deletion (DiGeorge/velocardiofacial syndrome), and Chr.1q21 deletion are the most commonly detected genomic disorders [7]. On the other hand, environmental factors, including maternal health conditions, like diabetes and hypertension, as well as exposure to harmful substances during pregnancy, can also affect kidney development [8]. These factors can result in various structural defects, such as renal agenesis, dysplasia, and ureteropelvic junction obstruction, which often require surgical intervention [9,10]. Therefore, early diagnosis through prenatal ultrasonography and genetic testing is critical in managing these conditions and preventing serious complications.

Although the initial diagnosis and management of CAKUT are well-defined, the longitudinal progression of the disease, particularly the changes in ultrasound findings over time and the factors determining the need for surgical intervention, has not been sufficiently studied in the pediatric population. This study aimed to investigate the relationship between genetic mutations and the need for ureterorenal surgery with prenatal, postnatal, and parental risk factors in children diagnosed with CAKUT.

## 2. Materials and Methods

This retrospective study was conducted on children diagnosed with CAKUT admitted to the Pediatric Nephrology Clinic of the Samsun Ondokuz Mayis University Faculty of Medicine Hospital between January 2020 and December 2022. This study adhered to the ethical regulations and principles specified in the Declaration of Helsinki and received approval from the Ethical Committee of Samsun Ondokuz Mayis University Faculty of Medicine (Date: 27 April 2023, Decision No. 2023/108). The requirement for obtaining informed consent was exempted by the Ethics Committee due to the retrospective design of this study.

### 2.1. Study Population

A total of 651 children diagnosed with CAKUT were retrospectively evaluated. In our clinic, specialists in neonatology screen all live births within the first week of life. Clinical geneticists screen patients with dysmorphology, and all diagnosed patients are then kept under clinical observation. Patients with incomplete diagnostic and follow-up data, those not followed up for at least six months after diagnosis, and cases of stillbirth or postnatal death were excluded from this study.

### 2.2. Data Collection and Definitions

Demographic and clinical data were collected using the hospital’s electronic information system and patient files. These data included demographic, clinical, and genetic findings such as the time of diagnosis, birth date, sex, prenatal ultrasound findings, type of anomaly, prematurity, birth weight, history of urinary tract infections, consanguinity, parental age, maternal gestational diabetes, hypertension during pregnancy, medications used by parents, maternal smoking habits, weight gain during pregnancy, parental kidney disease, postnatal ultrasound findings, postnatal type of anomaly, follow-up hydronephrosis, associated syndromes, and ureterorenal surgery.

Hydronephrosis affecting the renal pelvis or other urinary tract segments was classified as dilatation. Multicystic dysplastic kidneys were identified as structurally abnormal, with parenchymal cysts of varying sizes but without a renal pelvis.

### 2.3. Ultrasound Assessments

Detailed ultrasound assessments were integral to prenatal and postnatal evaluations in this study.

Obstetricians with at least five years of experience in prenatal ultrasonography conducted detailed ultrasound examinations to detect renal abnormalities, malformations, and markers of aneuploidy. Prenatal ultrasonography was utilized to identify renal pelvic dilatation and other renal abnormalities. Hydronephrosis was defined as a renal pelvis with an anteroposterior diameter over 4 mm before 28 weeks of gestation and over 7 mm after 28 weeks. Fetuses with signs of renal abnormalities on prenatal ultrasound were closely monitored.

Infants with prenatal indications of renal abnormalities were referred to the Pediatric Nephrology Department for postnatal ultrasound assessments. The first postnatal ultrasound was typically conducted 3–7 days after birth. Follow-up ultrasonographic evaluations were performed at 1, 2, 3, and 6 months post-birth to monitor the progression of hydronephrosis and other renal anomalies. These evaluations were performed by pediatric radiologists using high-resolution ultrasound machines to ensure accurate measurements of renal pelvis diameter, cortical thickness, and the presence of any cysts or structural abnormalities.

The criteria for postnatal hydronephrosis included an anteroposterior renal pelvic diameter greater than 10 mm. Additional ultrasonographic findings, such as parenchymal thinning, corticomedullary differentiation, and ureteral dilatation, were also documented.

### 2.4. Statistical Analysis

All data were analyzed using IBM SPSS Statistics for Windows, version 20.0 (IBM Corp., Armonk, NY, USA). Numerical data determined to be normally distributed based on the results of Kolmogorov–Smirnov tests are presented as mean ± standard deviation (SD) values. In contrast, non-normally distributed variables are presented as median (min–max) values. For comparisons between groups, Student’s *t*-test and Mann–Whitney U test were used according to the normality of the distribution. Categorical variables are presented as numbers and percentages, and inter-group comparisons were conducted using Chi-square and Fisher exact tests. Significance was accepted at *p* < 0.05 for all statistical analyses.

## 3. Results

### 3.1. Study Population

This study included 651 CAKUT patients with a mean diagnosis time of 22.2 ± 3.6 weeks of pregnancy. The majority were boys (60.5%). The rate of premature birth was 15.2%, and 42.2% had a positive history of urinary tract infections (UTIs). Consanguineous marriage was noted in 12% of the parents. Table 1 presents the demographic profiles of the patients and their parents.

### 3.2. Genetic Findings

Genetic mutations or syndromes were detected in 5.4% (*n* = 35) of the cases. The identified genetic abnormalities included a broad range of known genetic syndromes and mutations such as 10p deletion, Bardet–Biedel syndrome (*BBS9* gene exon 17 homozygous deletion and *BBS4* gene homozygous frameshift mutation c.210_213 del; p.(II70Metfs*5)), Bartter syndrome, Beckwith–Wiedemann syndrome, DiGeorge syndrome, Down syndrome, Fraser syndrome, Genitopatellar syndrome, Hajdu–Cheney syndrome, and Cornelia De Lange syndrome (Supplementary Table S1).

### 3.3. Surgical Interventions and Long-Term Outcomes

A substantial number of cases (24.6%) required surgical intervention. Long-term follow-ups revealed that approximately 4.1% of patients progressed to chronic kidney disease.

### 3.4. Comparison of Children with and without Genetic Mutations or Syndromes

Children with genetic mutations or syndromes had a lower mean neonatal birth weight compared to those without (2797.0 ± 688.1 vs. 3129.5 ± 609.9 g, *p* = 0.002) and a higher rate of prematurity (28.6% vs. 14.4%, *p* = 0.024). Additionally, maternal age (36.4 ± 7.2 vs. 33.2 ± 6.9 years, *p* = 0.006) and paternal age (39.8 ± 7.8 vs. 36.4 ± 7.3 years, *p* = 0.008) were higher in those with genetic mutations or syndromes. Other patient and parental characteristics did not show any significant association with the presence of genetic mutations or syndromes (Table 1).

### 3.5. Antenatal and Postnatal Ultrasound Findings

In the antenatal period, the incidence of polycystic kidney disease was higher in children with genetic mutations or syndromes compared to those without (5.7% vs. 0.3%, *p* = 0.035). In the postnatal period, the incidence of hydronephrosis was lower in children with genetic mutations or syndromes (40.0% vs. 69.8%, *p* < 0.001), whereas the incidence of abnormal ultrasound findings was higher (54.3% vs. 25.8%, *p* < 0.001) (Table 2).

Postnatally, the incidence of hydronephrosis and other anomalies increased (antenatal: 50.1% vs. postnatal: 68.2%, *p* < 0.001 for hydronephrosis; antenatal: 16.3% vs. postnatal: 27.3%, *p* < 0.001 for abnormal USG findings). The final ultrasound findings showed that the incidence of hypoplastic kidney (11.4% vs. 3.1%, *p* < 0.001) and polycystic kidney disease (25.7% vs. 2.9%, *p* < 0.001) were higher in children with genetic mutations or syndromes (Table 2).

In the 6-month follow-up of patients with hydronephrosis, improvement in the condition did not show a significant difference based on the presence of genetic mutations or syndromes (Supplementary Table S2).

### 3.6. Association of Ultrasound Findings With Surgical Intervention

A history of UTIs was higher in patients who underwent surgical intervention compared to those who did not (69.6% vs. 32.2%, *p* < 0.001). Other patient and parental characteristics did not significantly affect the requirement for surgery (Table 3).

In the antenatal period, hydronephrosis was more common in patients who required surgical intervention than in those who did not (70.2% vs. 43.5%, *p* < 0.001). This trend persisted in the postnatal period (84.5% vs. 62.9%, *p* < 0.001). However, the final ultrasound findings showed that the rate of hydronephrosis was lower in patients who underwent surgery compared to those who did not (30.4% vs. 45.5%, *p* < 0.001). In comparison, the rate of abnormal ultrasound findings was higher (67.7% vs. 39.8%, *p* < 0.001) (Table 4).

In both the antenatal and postnatal periods, the rate of multicystic dysplastic kidney was higher in patients who did not need surgical intervention compared to those who did (5.7% vs. 1.2%, *p* < 0.001 for antenatal period; 9.6% vs. 1.2%, *p* < 0.001 for postnatal period). Additionally, in the postnatal period, the rates of polycystic kidney disease and horseshoe kidney were higher in patients who did not need surgical intervention compared to those who did (3.9% vs. 0%, *p* < 0.001 for polycystic kidney disease; 2.9% vs. 0%, *p* < 0.001 for horseshoe kidney). During the final ultrasound period, patients requiring surgical intervention had higher rates of double collecting systems, ureterovesical junction narrowing, ureteropelvic junction narrowing, posterior urethral valves, vesicoureteral reflux, and ureterocele (Table 4).

### 3.7. Hydronephrosis Follow-Up

In the 6-month follow-up of patients with hydronephrosis, 20.6% showed improvement, 40.7% did not improve, and the remaining patients showed a reduction in hydronephrosis. The rate of reduction in hydronephrosis was higher in those who underwent surgical intervention compared to those who did not (55.8% vs. 31.2%, *p* < 0.001) (Supplementary Table S3).

In the 6-month follow-up, the rates of hydronephrosis did not show a significant difference between the groups with and without genetic mutations or syndromes (Supplementary Table S4).

### 3.8. Factors Associated With Genetic Mutations or Syndromes and Surgical Requirements

The potential risk factors linked to genetic mutations were identified as neonatal birth weight, prematurity, maternal and paternal age at childbirth, and postnatal ultrasound findings. These factors were included in the multivariate regression model. Multivariate analysis revealed that a one-gram decrease in neonatal birth weight increased the likelihood of a genetic mutation or syndrome by 1.02 times (odds ratio [OR] = 0.98, 95% confidence interval [CI] = 0.97–0.99, *p* < 0.001). Additionally, each one-year increase in maternal age at childbirth increased the likelihood of a genetic mutation or syndrome by 1.06 times (OR = 1.06, 95% CI = 1.01–1.11, *p* < 0.001). Postnatal abnormal ultrasound findings also increased the likelihood of a genetic mutation or syndrome by 2.62 times (OR = 2.62, 95% CI = 1.28–5.33, *p* < 0.001) (Table 5).

The potential risk factors associated with the need for surgery were identified as a history of prematurity and antenatal ultrasound findings. These factors were included in the multivariate regression model. The likelihood of requiring surgery was 4.85 times higher in those with a history of UTIs compared to those without (OR = 4.85, 95% CI = 3.26–7.21, *p* < 0.001), and 3.85 times higher in those with antenatal hydronephrosis findings compared to those with normal ultrasound results (OR = 3.85, 95% CI = 2.37–6.25, *p* < 0.001) (Table 5).

## 4. Discussion

This study represents a comprehensive analysis of prenatal and postnatal characteristics in children with CAKUT, examining genetic and parental factors, changes in ultrasound findings, and factors associated with surgical intervention. The key findings are as follows: (1) Neonatal birth weight, maternal age at childbirth, and postnatal abnormal ultrasound findings were identified as independent risk factors for genetic mutations or syndromes. (2) No significant association was found between the presence of genetic mutations or syndromes and the need for surgical intervention in CAKUT cases with varying genetic mutations. (3) The need for surgery was independently associated with a history of UTIs and the presence of antenatal hydronephrosis.

This study sheds light on the complex nature of CAKUT, contributing to approximately 20–30% of all prenatally detected anomalies and remaining a leading cause of kidney failure [11]. The etiology of CAKUT often involves unclear factors influenced by parental factors, pre-pregnancy and pregnancy conditions, intrauterine environments, and genetic components. The development of the mammalian kidney during embryonic stages involves reciprocal inductive events between the ureteric bud and metanephric mesenchyme [2]. Exposure to environmental risk factors or dysfunction in the genes that regulate this process can result in CAKUT [12]. The observed 5.4% rate of genetic mutations aligns with the literature suggesting that monogenic mutations constitute approximately 5–20% of cases [13,14]. Among the 27 identified genetic mutations or syndromes, the most frequently observed were neurofibromatosis type 1, tuberous sclerosis, Caroli syndrome, Turner syndrome, VACTERL syndrome, hyperphenylalaninemia, and compound heterozygote MTHFR. These anomalies have been previously identified as genetic factors affecting the kidneys [15,16,17,18,19]. A study conducted with data from diverse regions pointed out that the genetic mutations responsible for CAKUT may differ among various racial groups [6]. Therefore, the development of CAKUT and associated risk factors may vary according to ethnic group and region. In a comprehensive cohort study carried out in the United States, Asians and Hispanics were observed to have a higher risk of CAKUT compared to whites, whereas blacks showed a lower risk [20]. Additionally, CAKUT was significantly associated with lower gestational age, lower birth weight, known genetic disorders, and extrarenal anomalies [20]. In China, a study identified pathogenic variants in the *HNF1B*, *UMOD*, *NEK8*, and *BBS2* genes, whereas a study in India found 10 potentially harmful variants of uncertain significance in five genes (*FRAS1*, *TNXB*, *FREM2*, *SIX5*, and *CHD1L*) [21,22]. Nevertheless, these studies did not investigate the risk factors associated with the pathogenic variants. A study conducted in Korea identified seven single nucleotide variants that were either pathogenic or likely pathogenic in the *HNF1B*, *PAX2*, *EYA1*, *UPK3A*, and *FRAS1* genes. However, these pathogenic variants were not linked to factors such as preterm birth, low birth weight for gestational age, or oligohydramnios [23].

In the current study, while the mean age of both mothers and fathers was in the third decade, maternal age was identified as an independent risk factor for the presence of genetic mutations or syndromes. A previous study from Taiwan reported that the likelihood of CAKUT increases with advancing maternal age [24]. In the mentioned study, it was shown that maternal age under 20 reduces the likelihood of CAKUT by 1.38-fold (1/OR) (OR = 0.72), (95% CI = 0.55–0.93) compared to the 20–24 age range, while maternal age over 35 increases the likelihood by 1.28-fold (OR = 1.28), (95% CI = 1.14–1.44) [24]. In another study from Taiwan, the risk of CAKUT was found to be 2.29-fold higher in mothers aged 30–39 years compared to those under 20 years (OR = 2.29), (95% CI = 1.17–4.51) [14]. On the other hand, a study conducted in the Netherlands found that higher maternal age is linked to a reduced development of posterior urethral valves [25]. It has been demonstrated that advanced parental ages play a significant role in the occurrence of human germline mutations [26]. Studies have shown that germline mutations can cause CAKUT [15,27]. A previous study has shown that a father in his 20 s transmits approximately 25 de novo mutations that spontaneously occur in his sperm cells to his child, and this number doubles every 16.5 years [28]. These findings support the evidence that advancing parental age plays a significant role in genetic mutations. However, there may be specific mechanisms that cause maternal age to have a more significant impact than paternal age. Firstly, advanced maternal age is strongly associated with an increased risk of chromosomal abnormalities, such as trisomy 21 (Down syndrome). This is largely due to the higher likelihood of meiotic errors occurring during oocyte development as maternal age increases [29]. Secondly, as maternal age increases, the quality of eggs declines, leading to a higher incidence of genetic mutations. This is attributed to prolonged exposure to environmental factors and the natural aging process, which affects cellular mechanisms and increases the likelihood of chromosomal nondisjunction [30]. Finally, while advanced paternal age also contributes to an increased number of de novo mutations due to continuous spermatogenesis and cumulative DNA replication errors, the overall impact of maternal age on chromosomal integrity and egg quality tends to have a more immediate and pronounced effect on genetic outcomes in offspring [31].

Low neonatal birth weight was identified as another independent risk factor for genetic mutations or syndromes, consistent with findings from previous studies conducted in different regions [14,32,33]. This is probably related to maternal malnutrition and placental insufficiency, both of which are associated with low birth weight and prematurity. These conditions are linked to a reduced nephron count [34]. In the current literature, other parental health conditions and behaviors shown as potential risk factors for the development of CAKUT include the presence of gestational diabetes and hypertension, smoking, and medication use [3,35,36]. However, these confounding factors were not found to be associated with genetic mutations or syndromes. On the other hand, the rate of consanguineous marriage was relatively higher in cases with genetic mutations or syndromes. This may contribute to the genetic factors involved in CAKUT [37].

Ultrasound evaluations can guide the identification of patients at high risk for genetic mutations or syndromes. The frequency of polycystic kidney disease was notably higher in cases with genetic mutations or syndromes during both the antenatal and postnatal periods. Polycystic kidney disease typically follows an autosomal dominant or autosomal recessive inheritance pattern, heightening the relevance of genetic testing in these cases [37]. Other renal anomalies such as hydronephrosis or renal agenesis are often the result of complex genetic and environmental interactions [38]. On the other hand, the most frequently observed anomaly was hydronephrosis, consistent with the current literature [38,39]. Although hydronephrosis comprised about 30% of the cases with genetic mutations or syndromes, it was not identified as a significant risk factor. This may be associated with the improvement or reduction in hydronephrosis observed in a substantial number of cases. Therefore, the timing of ultrasound evaluations may hold prognostic importance.

A study conducted on CAKUT cases in Brazil indicated that elevated antenatal anterior–posterior renal pelvic diameter are an independent predictor for the necessity of postnatal surgical intervention [9]. In the current study, the presence of antenatal hydronephrosis was an independent predictor of the need for uretero-renal surgery. Furthermore, the frequency of improvement in hydronephrosis during the initial 6-month follow-up period was lower in cases requiring uretero-renal surgery compared to those that did not require surgery. Prenatal hydronephrosis represents the diverse range of pathologies observed within CAKUT and may be the initial indicator of either a transient issue or a more serious underlying condition [3]. In a meta-analysis study on antenatal hydronephrosis, pathology was confirmed in 36% of patients during postnatal evaluation, and pathology was detected in 88% of cases with severe antenatal hydronephrosis [40]. In these cases, postnatal follow-up may uncover additional pathologies, such as uretero-pelvic junction stenosis or vesicoureteral reflux [41]. In this study, genetic mutations or syndromes were not associated with the need for surgery. Still, final diagnoses such as vesicoureteral reflux, ureteropelvic junction narrowing, posterior urethral valves, and ureterocele were associated. The high frequency of abnormal findings in the final diagnosis and the low success rate of conservative management point to the aggressive nature of CAKUT and the frequent need for surgical intervention. A study conducted in Switzerland reported that impaired kidney function and postnatal bilateral kidney anomalies are potential predictors for surgical intervention [42]. These findings underscore the importance of early and precise prenatal ultrasound measurements in identifying neonates who are at a higher risk and may benefit from timely surgical management postpartum.

The generalizability of our results could be influenced by the genetic and environmental diversity among different populations. To address this, future research should include multi-center studies with larger, diverse cohorts to validate our findings. Additionally, investigating regional environmental factors and parental health conditions in more detail could provide further insights into the etiology of CAKUT. We believe that expanding our research to include diverse populations and collaborating with international research groups will enhance our understanding of CAKUT and improve the generalizability of our results. This will ultimately lead to better diagnostic and therapeutic strategies tailored to different populations.

This study had several important limitations. Firstly, it was conducted exclusively at a single tertiary care center, which limits the generalizability of its findings to the broader population. Secondly, the data collection relied on the retrospective analysis of patient records, potentially missing some important risk factors associated with CAKUT. Thirdly, infants with normal antenatal ultrasound results and those without clinical symptoms postnatally in the first six months were classified as normal and excluded from this study. This exclusion may have led to an under-representation of children with CAKUT, as the most clinically significant cases often manifest symptoms within the first six months of life [43]. Additionally, the follow-up period for assessing outcomes was relatively short. CAKUT is a condition with significant long-term implications, including the progression to chronic kidney disease, end-stage renal disease, and other complications such as hypertension and UTIs [44,45]. Long-term follow-up is essential to understand the full spectrum of outcomes and to identify early predictors of adverse long-term consequences. Studies with extended follow-up periods have shown that children with CAKUT are at increased risk of developing chronic kidney disease and end-stage renal disease later in life [11,46,47]. The progression of renal dysfunction can vary significantly among individuals, necessitating long-term monitoring to identify those who may benefit from early interventions. Future studies should incorporate extended follow-up periods to capture these long-term outcomes and provide a more comprehensive understanding of the disease progression in CAKUT patients.

## 5. Conclusions

The complex, multifactorial nature of CAKUT is underscored by the fact that many cases do not involve genetic anomalies. Approximately half of the patients exhibited hydronephrosis during the antenatal period, while one-third had normal ultrasound findings. However, there was a notable increase in abnormal ultrasound findings during the postnatal period, highlighting the importance of ongoing ultrasound monitoring in the first six months after birth for suspected CAKUT cases. Low birth weight, advanced parental age, and abnormal postnatal ultrasound results should be considered when identifying patients at risk for genetic mutations or syndromes. Moreover, patients with antenatal hydronephrosis and a history of postnatal UTIs may require early surgical intervention.

## Figures and Tables

**Table 1:** Demographic profiles of patients and their parents in children diagnosed with congenital anomalies of the kidney and urinary tract.

Variables	All Population	Genetic Mutations/Syndromes		*p*-Value
		No	Yes	
	n = 651	n = 616	n = 35	
Time of diagnosis, week of pregnancy	22.2 ± 3.6	22.2 ± 3.6	21.1 ± 2.8	0.228
Gender, n (%)				
Girls	257 (39.5)	238 (38.6)	19 (54.3)	0.065
Boys	394 (60.5)	378 (61.4)	16 (45.7)	
Neonatal birth weight, grams	3111.6 ± 618.4	3129.5 ± 609.9	2797.0 ± 688.1	0.002 *
Prematurity, n (%)	99 (15.2)	89 (14.4)	10 (28.6)	0.024 *
History of UTIs, n (%)	270 (41.5)	260 (42.2)	10 (28.6)	0.111
Parental consanguinity, n (%)	79 (12.1)	72 (11.7)	7 (20.0)	0.143
Maternal				
Age at childbirth, years	33.4 ± 6.9	33.2 ± 6.9	36.4 ± 7.2	0.006 *
Weight gain during pregnancy, kg	12.0 (10.0–15.0)	12.0 (10.0–15.0)	14.0 (10.0–16.0)	0.224
Gestational diabetes, n (%)	42 (6.5)	41 (6.7)	1 (2.9)	0.374
Pregnancy hypertension, n (%)	40 (6.1)	38 (6.2)	2 (5.7)	0.913
Medication use, n (%)	189 (29.0)	183 (29.7)	6 (17.1)	0.112
Smoking, n (%)	90 (13.8)	85 (13.8)	5 (14.3)	0.935
Family history of kidney disease	145 (22.3)	136 (22.1)	9 (25.7)	0.615
Paternal				
Age at childbirth, years	36.6 ± 7.4	36.4 ± 7.3	39.8 ± 7.8	0.008 *
Hypertension, n (%)	46 (7.1)	43 (7.0)	3 (8.6)	0.721
Medication use, n (%)	89 (13.7)	83 (13.5)	6 (17.1)	0.539
Smoking, n (%)	357 (54.8)	343 (55.7)	14 (40.0)	0.070
Family history of kidney disease	143 (22.0)	135 (21.9)	8 (22.9)	0.896

Categorical variables were shown as numbers and percentages. Numerical variables are mean ± SD or median (IQR). * *p*-value < 0.05 shows statistical significance. UTIs, urinary tract infections.

**Table 2:** Antenatal and postnatal USG findings in children diagnosed with congenital anomalies of the kidney and urinary tract.

Variables	All	Genetic Mutation/Syndrome		*p*-Value
	Population	No	Yes	
	n = 651	n = 616	n = 35	
Antenatal USG, n (%)				
Normal	219 (33.6)	202 (32.8)	17 (48.6)	0.069
Hydronephrosis	326 (50.1)	315 (51.1)	11 (31.4)	
Abnormal findings	106 (16.3)	99 (16.1)	7 (20.0)	
Agenesis	39 (6.0)	37 (6.0)	2 (5.7)	0.035 *
Double collecting system	4 (0.6)	4 (0.6)	-	
Hypoplastic kidney	15 (2.3)	15 (2.4)	-	
Ectopic kidney	6 (0.9)	6 (1.0)	-	
Ureteropelvic junction narrowing	2 (0.3)	2 (0.3)	-	
Posterior urethral valves	2 (0.3)	2 (0.3)	-	
Multicystic dysplastic kidney	30 (4.6)	28 (4.5)	2 (5.7)	
Polycystic kidney disease	4 (0.6)	2 (0.3)	2 (5.7)	
Horseshoe kidney	4 (0.6)	3 (0.5)	1 (2.9)	
Postnatal USG, n (%)				
Normal	29 (4.5)	27 (4.4)	2 (5.7)	<0.001 *
Hydronephrosis	444 (68.2)	430 (69.8)	14 (40.0)	
Abnormal findings	178 (27.3)	159 (25.8)	19 (54.3)	
Agenesis	45 (6.9)	44 (7.1)	1 (2.9)	<0.001 *
Double collecting system	16 (2.5)	16 (2.6)	-	
Hypoplastic kidney	20 (3.1)	17 (2.8)	3 (8.6)	
Ectopic kidney	14 (2.2)	13 (2.1)	1 (2.9)	
Multicystic dysplastic kidney	49 (7.5)	43 (7.0)	6 (17.1)	
Vesicoureteral reflux	1 (0.2)	1 (0.2)	-	
Polycystic kidney disease	19 (2.9)	12 (1.9)	7 (20.0)	
Horseshoe kidney	14 (2.2)	13 (2.1)	1 (2.9)	
Final USG, n (%)				
Normal	75 (11.5)	73 (11.9)	2 (5.7)	0.080
Hydronephrosis	272 (41.8)	262 (42.5)	10 (28.6)	
Abnormal findings	304 (46.5)	281 (45.6)	23 (65.7)	
Agenesis	45 (6.9)	44 (7.1)	1 (2.9)	<0.001 *
Double collecting system	22 (3.4)	22 (3.6)	-	
Hypoplastic kidney	23 (3.5)	19 (3.1)	4 (11.4)	
Ectopic kidney	15 (2.3)	14 (2.3)	1 (2.9)	
Ureterovesical junction narrowing	3 (0.5)	3 (0.5)	-	
Ureteropelvic junction narrowing	32 (4.9)	31 (5.0)	1 (2.9)	
Posterior urethral valves	12 (1.8)	11 (1.8)	1 (2.9)	
Multicystic dysplastic kidney	48 (7.4)	44 (7.1)	4 (11.4)	
Vesicoureteral reflux	55 (8.4)	54 (8.8)	1 (2.9)	
Polycystic kidney disease	27 (4.1)	18 (2.9)	9 (25.7)	
Horseshoe kidney	16 (2.5)	15 (2.4)	1 (2.9)	
Ureterocele	6 (0.9)	6 (1.0)	-	

Categorical variables were shown as numbers and percentages. * *p*-value < 0.05 shows statistical significance. USG, ultrasonography.

**Table 3:** Demographic profiles of patients and their parents based on the need for surgery in children diagnosed with congenital anomalies of the kidney and urinary tract.

Variables	Uretero-Renal Surgery		*p*-Value
	No	Yes	
	n = 490	n = 161	
Time of diagnosis, week of pregnancy	22.0 ± 3.4	22.5 ± 4.0	0.274
Gender, n (%)			
Girls	198 (40.4)	59 (36.6)	0.397
Boys	292 (59.6)	102 (63.4)	
Neonatal birth weight, grams	3102.8 ± 624.0	3138.4 ± 602.2	0.424
Prematurity, n (%)	78 (15.9)	21 (13.0)	0.378
History of UTI, n (%)	158 (32.2)	112 (69.6)	<0.001 *
Parental consanguinity, n (%)	60 (12.2)	19 (11.8)	0.881
Maternal			
Age at childbirth, years	33.4 ± 6.9	33.5 ± 7.2	0.954
Weight gain during pregnancy, kg	12.0 (10.0–15.0)	12.0 (10.0–16.0)	0.225
Gestational diabetes, n (%)	33 (6.7)	9 (5.6)	0.608
Pregnancy hypertension, n (%)	31 (6.3)	9 (5.6)	0.736
Medication use, n (%)	144 (29.4)	45 (28.0)	0.727
Smoking, n (%)	64 (13.1)	26 (16.1)	0.325
Family history of kidney disease	108 (22.0)	37 (23.0)	0.803
Paternal			
Age at childbirth, years	36.6 ± 7.3	36.7 ± 7.7	0.746
Hypertension, n (%)	36 (7.3)	10 (6.2)	0.626
Medication use, n (%)	67 (13.7)	22 (13.7)	0.998
Smoking, n (%)	269 (54.9)	88 (54.7)	0.958
Family history of kidney disease	109 (22.2)	34 (21.1)	0.764
Genetic mutation /syndrome, n (%)	27 (5.5)	8 (5.0)	0.792

Categorical variables were shown as numbers and percentages. Numerical variables are mean ± SD or median (IQR). * *p*-value < 0.05 shows statistical significance. UTI, urinary tract infection.

**Table 4:** Antenatal and postnatal USG findings based on the need for surgery in children diagnosed with congenital anomalies of the kidney and urinary tract.

Variables	Uretero-Renal Surgery		*p*-Value
	No	Yes	
	n = 490	n = 161	
Antenatal USG, n (%)			
Normal	192 (39.2)	27 (16.8)	<0.001 *
Hydronephrosis	213 (43.5)	113 (70.2)	
Abnormal findings	85 (17.3)	21 (13.0)	
Agenesis	30 (6.1)	9 (5.6)	<0.001 *
Double collecting system	3 (0.6)	1 (0.6)	
Hypoplastic kidney	11 (2.2)	4 (2.5)	
Ectopic kidney	5 (1.0)	1 (0.6)	
Ureteropelvic junction narrowing	-	2 (1.2)	
Posterior urethral valves	-	2 (1.2)	
Multicystic dysplastic kidney	28 (5.7)	2 (1.2)	
Polycystic kidney disease	4 (0.8)	-	
Horseshoe kidney	4 (0.8)	-	
Postnatal USG, n (%)			
Normal	25 (5.1)	4 (2.5)	<0.001 *
Hydronephrosis	308 (62.9)	136 (84.5)	
Abnormal findings	157 (32.0)	21 (13.0)	
Agenesis	41 (8.4)	10 (6.2)	<0.001 *
Double collecting system	9 (1.8)	7 (4.3)	
Hypoplastic kidney	17 (3.5)	3 (1.9)	
Ectopic kidney	13 (2.7)	1 (0.6)	
Multicystic dysplastic kidney	47 (9.6)	2 (1.2)	
Vesicoureteral reflux	-	1 (0.6)	
Polycystic kidney disease	19 (3.9)	-	
Horseshoe kidney	14 (2.9)	-	
Final USG, n (%)			
Normal	72 (14.7)	3 (1.9)	<0.001 *
Hydronephrosis	223 (45.5)	49 (30.4)	
Abnormal findings	195 (39.8)	109 (67.7)	
Agenesis	39 (8.0)	6 (3.7)	<0.001 *
Double collecting system	12 (2.4)	10 (6.2)	
Hypoplastic kidney	21 (4.3)	2 (1.2)	
Ectopic kidney	13 (2.7)	2 (1.2)	
Ureterovesical junction narrowing	-	3 (1.9)	
Ureteropelvic junction narrowing	-	32 (19.9)	
Posterior urethral valves	1 (0.2)	11 (6.8)	
Multicystic dysplastic kidney	45 (9.2)	3 (1.9)	
Vesicoureteral reflux	17 (3.5)	38 (23.6)	
Polycystic kidney disease	27 (5.5)	-	
Horseshoe kidney	15 (3.1)	1 (0.6)	
Ureterocele	-	6 (3.7)	

Categorical variables were shown as numbers and percentages. * *p*-value < 0.05 shows statistical significance. USG, ultrasonography.

**Table 5:** Independent factors associated with genetic mutation/syndrome and need for surgery.

Variables	Univariable Regression			Multivariable Regression		
	OR	95% CI	*p*-Value	OR	95% CI	*p*-Value
Genetic mutations/syndromes						
Neonatal birth weight	0.98	0.97–0.99	0.002 *	0.98	0.97–0.99	0.019 *
Prematurity	2.37	1.10–5.10	0.024 *	-	-	-
Maternal age at childbirth	1.07	1.02–1.12	0.006 *	1.06	1.01–1.11	0.024 *
Paternal age at childbirth	1.06	1.02–1.11	0.008 *	-	-	-
Postnatal USG						
Normal	ref			ref		
Hydronephrosis	0.42	0.19–0.90	0.028 *	0.46	0.11–1.12	0.084
Abnormal Finding	3.41	1.71–6.80	<0.001 *	2.62	1.28–5.33	0.008 *
				Nagelerke *R*^2^ = 0.38		
Need of uretero-renal surgery						
History of UTIs	4.80	3.27–7.06	<0.001 *	4.85	3.26–7.21	<0.001 *
Antenatal USG						
Normal	ref					
Hydronephrosis	3.77	2.38–5.99	<0.001 *	3.85	2.37–6.25	<0.001 *
Abnormal findings	1.76	0.94–3.28	0.077	1.85	0.96–3.55	0.070
				Nagelerke *R*^2^ = 0.32		

* *p*-value < 0.05 shows statistical significance. CI, confidence interval; OR, odds ratio; ref, reference, USG, ultrasonography; UTIs, urinary tract infections.

## Data Availability

The data that support the findings of this study are available upon request from the corresponding author.

## Supplementary Materials

The following supporting information can be downloaded at: https://www.mdpi.com/article/10.3390/biomedicines12081798/s1, Table S1: The genetic mutations and associated syndromes of children with CAKUT; Table S2: Changes in USG results according to genetic mutation or syndrome in patients who develop hydronephrosis; Table S3: The relationship between changes in USG results and the need for surgery in the follow-up of patients who develop hydronephrosis; Table S4: Changes in USG results according to genetic mutation or syndrome in patients who develop hydronephrosis.
