# Supplementary material for: Genetic and Clinical Factors Influencing Congenital Anomalies of the Kidney and Urinary Tract in Children: Insights from Prenatal and Postnatal Assessments
Source: Biomedicines. 2024 Aug 8;12(8):1798. doi: 10.3390/biomedicines12081798 (PMC11351149; doi:10.3390/biomedicines12081798)
Supplement: Supplementary file 1 [file biomedicines-12-01798-s001.zip › biomedicines-3126880-supplementary.pdf]

Supplementary Table 1. The genetic mutations and associated syndromes of children with CAKUT

Variables	All population	Uretero-renal surgery		*P*-value
		No	Yes	
	n = 651	n = 490	n = 161	
Genetic mutation / syndrome, n (%)	35 (5.4)	27 (5.5)	8 (5.0)	0.792
10p Deletion	1 (0.15)	-	1 (0.6)	
Bardet-Biedel Syndrome (BBS9 gene, Exon 17 Homozygous Deletion)	1 (0.15)	1 (0.2)	-	
Bardet-Biedel Syndrome (Homozygous frameshift mutation c.210_213 del in BBS4 gene; p.(II70Metfs*5)	1 (0.15)	1 (0.2)	-	
Bartter Syndrome	1 (0.15)	1 (0.2)	-	
Beckwith-Widemann Syndrome	1 (0.15)	-	1 (0.6)	
Deletion in BRCA2 gene	1 (0.15)	-	1 (0.6)	
Caroli Syndrome (Congenital Hepatic Fibrosis)	2 (0.3)	1 (0.2)	-	
Cornelia De Lange Syndrome	1 (0.15)	1 (0.2)	-	
DiGeorge Syndrome	1 (0.15)	1 (0.2)	-	
Down Syndrome	1 (0.15)	1 (0.2)	-	
Fraser Syndrome	1 (0.15)	-	1 (0.6)	0.999
Genito-Patellar Syndrome (Midline Defect+Corpus Callosum Agenesis+Anal Atresia+Patient with Tracheostomy)	1 (0.15)	-	1 (0.6)	
Hajdu-Cheney Syndrome (NOTCH2)	1 (0.15)	1 (0.2)	-	
Hyperphenylalaninemia, Compound Heterozygote MTHFR	2 (0.3)	2 (0.4)	-	
Mitochondrial Disease, CDG, Factor 13 Mutation Heterozygote, MTHFR Compound Heterozygote Mutation, PAI1 Heterozygote Mutation, Branchiootorenal Syndrome 2, SIX5 gene with c.919G>A p.(Ala307Thr) heterozygous VUS, Nephronophthisis 14, ZNF423 gene with c.2812C>G p.(Gln938Glu) heterozygous VUS	1 (0.15)	1 (0.2)	-	

MTHFR C677T Homozygous, PAI-1 4G Homozygous Mutations, Carnitine Deficiency	1 (0.15)	-	1 (0.6)	
MTHFR Gene Mutation Homozygous	1 (0.15)	1 (0.2)	-	
Neurofibromatosis Type 1	3 (0.5)	3 (0.6)	-	
Partial Trisomy 2q	1 (0.15)	-	1 (0.6)	
PKD1 gene with c.12875G>A p.(Arg4292Gln) heterozygous VUS 2139762	1 (0.15)	1 (0.2)	-	
PKD1 gene with c.4373C>T p.(Ala1458Val) heterozygous missense VUS, ZNF423 gene with c.2972C>T p.(Ala991Val) heterozygous VUS (Nephronophthisis 14)	1 (0.15)	1 (0.2)	-	
PKD1 gene with Exon 1,2,3,4 Heterozygous Deletion	1 (0.15)	1 (0.2)	-	
PKD2 gene with c.1837C>T p.(Gln613*) heterozygous nonsense pathogenic	1 (0.15)	1 (0.2)	-	
Prune Belly Syndrome	1 (0.15)	-	1 (0.6)	
Tuberous Sclerosis	3 (0.5)	3 (0.6)	-	
Turner Syndrome (45,X0)	2 (0.3)	2 (0.4)	-	
VACTERL Syndrome	2 (0.3)	-	1 (0.6)	

Categorical variables were shown as number and percentages.

Supplementary Table 2. Changes in USG results according to genetic mutation or syndrome in patients who develop hydronephrosis

Follow-up USG findings	Genetic mutation / syndrome		*P*-value
	No<br>n = 438	Yes<br>n = 14	
1st month			
Normal	7 (1.6)	-	0.636
Hydronephrosis regression	11 (2.5)	1 (7.1)	
Hydronephrosis stable	408 (93.2)	13 (92.9)	
Hydronephrosis progression	12 (2.7)	-	
2nd months			
Normal	9 (2.1)	-	0.521
Hydronephrosis regression	103 (23.5)	5 (35.7)	
Hydronephrosis stable	260 (59.4)	6 (42.9)	
Hydronephrosis progression	66 (15.1)	3 (21.4)	
3rd months			
Normal	39 (8.9)	2 (14.3)	0.860
Hydronephrosis regression	165 (37.7)	5 (35.7)	
Hydronephrosis stable	201 (45.9)	6 (42.9)	
Hydronephrosis progression	33 (7.5)	1 (7.1)	
6th months			
Normal	91 (20.8)	2 (14.3)	0.126
Hydronephrosis regression	130 (29.7)	6 (42.9)	
Hydronephrosis stable	200 (45.7)	4 (28.6)	
Hydronephrosis progression	17 (3.9)	2 (14.3)	
Follow-up hydronephrosis			
Improved	91 (20.8)	2 (14.3)	0.778
Not improved	179 (40.9)	5 (35.7)	
Decreased	168 (38.4)	7 (50.0)	

Categorical variables were shown as number and percentages. * P-value <0.05 shows statistical significance. USG, ultrasonography

Supplementary Table 3. The relationship between changes in USG results and the need for surgery in the follow-up of patients who develop hydronephrosis.

Follow-up USG findings	All population n = 452	Uretero-renal surgery		*P*-value
		No n = 314	Yes n = 138	
1st month				
Normal	7 (1.5)	5 (1.6)	2 (1.4)	0,058
Hydronephrosis regression	12 (2.7)	9 (12.9)	3 (2.2)	
Hydronephrosis stable	421 (93.1)	296 (94.3)	125 (90.6)	
Hydronephrosis progression	12 (2.7)	4 (1.3)	8 (5.8)	
2nd months				
Normal	9 (2.0)	8 (2.5)	1 (0.7)	<0.001*
Hydronephrosis regression	108 (23.9)	82 (26.1)	26 (18.8)	
Hydronephrosis stable	266 (58.8)	194 (61.8)	72 (52.2)	
Hydronephrosis progression	69 (15.3)	30 (9.6)	39 (28.3)	
3rd months				
Normal	41 (9.1)	35 (11.1)	6 (4.3)	<0.001*
Hydronephrosis regression	170 (37.6)	100 (31.8)	70 (50.7)	
Hydronephrosis stable	207 (45.8)	163 (51.9)	44 (31.9)	
Hydronephrosis progression	34 (7.5)	16 (5.1)	18 (13.0)	
6th months				
Normal	93 (20.6)	74 (23.6)	19 (13.8)	<0.001*
Hydronephrosis regression	136 (30.1)	66 (21.0)	70 (50.7)	
Hydronephrosis stable	204 (45.1)	160 (51.0)	44 (31.9)	
Hydronephrosis progression	19 (4.2)	14 (4.5)	5 (3.6)	
Follow-up hydronephrosis				
Improved	93 (20.6)	74 (23.6)	19 (13.8)	<0.001*
Not improved	184 (40.7)	142 (45.2)	42 (30.4)	
Decreased	175 (38.7)	98 (31.2)	77 (55.8)	

Categorical variables were shown as number and percentages. * P-value <0.05 shows statistical significance. USG, ultrasonography

Supplementary Table 4. Changes in USG results according to genetic mutation or syndrome in patients who develop hydronephrosis

Follow-up USG findings	Genetic mutation / syndrome		*P*-value
	No<br>n = 438	Yes<br>n = 14	
1st month			
Normal	7 (1.6)	-	0.636
Hydronephrosis regression	11 (2.5)	1 (7.1)	
Hydronephrosis stable	408 (93.2)	13 (92.9)	
Hydronephrosis progression	12 (2.7)	-	
2nd months			
Normal	9 (2.1)	-	0.521
Hydronephrosis regression	103 (23.5)	5 (35.7)	
Hydronephrosis stable	260 (59.4)	6 (42.9)	
Hydronephrosis progression	66 (15.1)	3 (21.4)	
3rd months			
Normal	39 (8.9)	2 (14.3)	0.860
Hydronephrosis regression	165 (37.7)	5 (35.7)	
Hydronephrosis stable	201 (45.9)	6 (42.9)	
Hydronephrosis progression	33 (7.5)	1 (7.1)	
6th months			
Normal	91 (20.8)	2 (14.3)	0.119
Hydronephrosis regression	130 (29.7)	6 (42.9)	
Hydronephrosis stable	200 (45.7)	4 (28.6)	
Hydronephrosis progression	17 (3.9)	2 (14.3)	
Follow-up hydronephrosis			
Improved	91 (20.8)	2 (14.3)	0.778
Not improved	179 (40.9)	5 (35.7)	
Decreased	168 (38.4)	7 (50.0)	

Categorical variables were shown as number and percentages. * P-value <0.05 shows statistical significance. USG, ultrasonography
